# Impact of Nutritional and Environmental Factors on Inflammation, Oxidative Stress, and the Microbiome

**DOI:** 10.1155/2018/5606845

**Published:** 2018-07-31

**Authors:** Gang Liu, Yan Huang, Lidong Zhai

**Affiliations:** ^1^Laboratory of Animal Nutritional Physiology and Metabolic Process, Key Laboratory of Agro-Ecological Processes in Subtropical Region, Institute of Subtropical Agriculture, Chinese Academy of Sciences, National Engineering Laboratory for Pollution Control and Waste Utilization in Livestock and Poultry Production, Changsha, Hunan 410125, China; ^2^Department of Animal Science, Division of Agriculture, University of Arkansas, Fayetteville, AR72701, USA; ^3^Tianjin Medical University, Tianjin 300070, China

Studies suggest that active inflammatory response and oxidative stress are the most prominent symptoms suffered by patients with metabolic diseases. Meanwhile, the gut hosts a complex community of microorganisms which are highly associated with human physiology, metabolism, and immune status; the effect of gut microbiota in health and diseases becomes clear.

Growing evidence indicates that nutrients and environmental factors are tightly associated with the generation of reactive oxygen species (ROS) and reactive nitrogen species (RNS), oxidative stress, endoplasmic reticulum stress, and gut microbiome. Meanwhile, nutritional factors such as certain natural compounds and nutraceuticals may protect cells from oxidative/endoplasmic reticulum stress and thus ameliorate oxidative/endoplasmic reticulum stress-related diseases via changing of the microbiota. In this way, nutritional factors or molecules perform a vital function in repairing metabolic disorders that result from oxidative/endoplasmic reticulum stress. However, the detailed mechanisms underlying the role of environmental and nutritional factors on regulation of inflammation, oxidative/endoplasmic reticulum stress, and the microbiome in chronic diseases remain largely unexploited and unclear.

In our special issue, opened for 7 months from Sep 2017 to March 2018, focus on the evidences of recent researches related to natural compounds and environmental factors in regulation of oxidative/endoplasmic reticulum stress.

Maternal obesity or overnutrition may have a long-term effect on the health of offspring. L. Montalvo-Martínez et al. reviewed the recent advances on the effects of high-calorie nutrient fetal programming on central and peripheral inflammation and the behaviors of offspring addiction. Maternal overnutrition can cause neuronal gene expression programs to be set in the fetus. Selective intake of a diet rich in fats and sugars at key stages recruits markers of central and peripheral inflammatory cell types, thereby disrupting energy-sensing and behavioral pathways, increasing susceptibility, and exhibiting abnormal behavior similar to addiction. Through selective epigenetic activation in fetal programming, the addiction phenotype may be inherited transgenerationally.

9,10-Phenanthrenequinone (9,10-PQ) is a terpene molecule found in a large amount of air pollution in diesel exhaust particles (DEP) and has been shown to produce cytotoxicity both in vitro and in vivo. M. Yang et al. reviewed the latest research progress on 9,10-PQ and summarized that 9,10-PQ plays a key role in the development of cytotoxicity through the generation of reactive oxygen species (ROS) through redox cycling, with particular emphasis on the mechanisms that may be involved. Understanding the potential cellular mechanisms involved in cytotoxicity may allow the development of therapeutics aimed at targeting specific molecules that are significantly implicated in 9,10-PQ-induced ROS toxicity.

B. Mkhize et al. did an interesting cross-sectional study on the use of prealbumin as a tool for nutritional assessment in adults coinfected with HIV and intestinal helminth parasites in KwaZulu-Natal, South Africa. They found that, in the presence or absence of inflammation in various BMI categories, prealbumin can distinguish between inflammation-induced albumin reduction and real malnutrition in adults individually or coinfected with HIV and intestinal worms, indicated by elevated and normal C-reactive protein (CRP) levels. Despite the small sample size of this experiment, it may not be possible to confirm the significant correlation between BMI, prealbumin, and albumin changes. A deep sampling study with a random sample design and a large sample size longitudinal queue can be considered.

S. Li et al. investigated the effect of cyclic tensile strain (CTS) on the function of Schwann cells (SCs) derived from the sciatic nerves of the newly born rat. The number and proliferation of SCs increase and the SCs display strong polarity after 5 CTS. The results also showed 5% CTS reduced netrin gene expression and increased NGF, GDNF, and Slit-2 gene expression (P<0.05). These indicate that CTS may lead to the effective stimulation of SCs and cytoskeleton remodeling due to a related inducement in the upregulation of nerve-oriented factors expression.

Diacetyl is a flavoring that imparts buttery taste to food; however, the intake may be toxic. L. D. L. Jedlicka et al. performed a target metabolomics analysis of male and female C57/B1 mice with oral diacetyl by UPLC-MS/MS using Ultra Performance Liquid Chromatography with Triple Quadrupole Mass Spectrometry (UPLC-MS/MS). Most tests showed differences between control and treatment groups and between genders, indicating that sex hormones are involved in the regulation of the normal metabolic profile and the response to metabolite diseases.

An interesting study by S. Wang et al. described that this research aimed to investigate the effect of the bioactive substance secreted by mesenchymal stem cells (MSC-CM) on vascular calcification. The vascular smooth muscle cell (VSMC) calcification model was induced by *β*-glycerophosphate (*β*-GP) and treated by MSC-CM. The calcium deposition, intracellular calcium contents, alkaline phosphatase (ALP) activity, and the expression of specific-osteogenic markers, inflammatory cytokines, and apoptosis-associated genes/proteins of VSMCs were assessed. Treatment with MSC-CM significantly reduced calcium deposition seen on alizarin red and Von Kossa staining and intracellular calcium contents, alkaline phosphatase (ALP) activity, and the expression of specific-osteogenic markers. Furthermore, MSC-CM also suppressed the expression of inflammatory cytokines and apoptosis-associated genes/proteins. MSC-CM could be a potential novel clinical treatment of vascular calcification.

Oxidative stress (OS) plays a key role in the pathogenesis and development of IBD. G. Guan and S. Lan reviewed the production of reactive oxygen species and antioxidant defense mechanisms in the gastrointestinal tract and the role of OS in the pathogenesis and development of labeled IBD, demonstrating various pathways associated with OS and genetic susceptibility and mucosal immune responses. Current clinical studies and experimental anti-IBD therapies, especially those that include naturally occurring antioxidants, correlate with positive IBD and colorectal cancer patient results. Antioxidant activity characterized by high levels of specificity may be the basis for the development of clinical treatment and relapsed IBD patients. With the introduction of new antioxidant-enhancing interventions, coupled with the management of traditional medicines, it is expected that IBD patients will benefit from significantly more favorable results from further clinical trials.

A review article written by Md. A. K. Azad et al. focused on gut microbiota, its modulation with the change in diet and nutrients, and the adverse effects of gut modulation on host health while gut modulation actions of probiotics (*Lactobacillus*,* Bifidobacterium*, and other bacteria species, mainly* Escherichia coli* and* Enterococcus*) in various diseases models. Probiotic bacterial species are reported to have significant roles, like improvement in digestion and immune system, synthesis of B vitamin, promotion of angiogenesis, and nerve system along with alteration in several degenerative diseases, like cardiovascular disease, cancer, malignancy, type 2 diabetes mellitus, obesity, colitis, asthma, and psychiatric and inflammatory disorders.

A. Emamverdian et al. described silicon (Si) mechanisms to ameliorate heavy metal stress in plants. They have shown that Si involved in the alleviation process in plants exposed to abiotic stresses and heavy metals in some important mechanisms, including (1) reduction of heavy metal uptake by plant, (2) change in pH value in soil and plant culture, (3) formation of Si heavy metal, and (4) stimulation of enzyme activities. Through these mechanisms Si alleviated and reduced uptake heavy metal and its transport in plants, changed pH of soil and growth medium, decreased the toxicity, and increased antioxidant activities. These findings could be a resourceful reference for future research.

T. Liu et al. described a study that aimed to investigate the effects of leucine supplementation in premating diet on the reproductive performance, maternal antioxidative capability, and immune function in primiparous rats. Leucine supplementation decreased significantly within-litter birth weight variation and improved the embryo distribution uniformity and the number of implantation sites in uterine compared with control group. Moreover, leucine treatment showed the upregulated effect on the expression of LHR, CYP19A1, and VEGFA but decreased the expression of Mucin-1. In addition, leucine enhanced the maternal antioxidant capacity and immune function. Those results demonstrated that leucine supplementation may improve the reproductive performance by improving oxidative and immune status.

S. Liu et al. described that the antioxidative function of alpha-ketoglutarate (AKG) and its applications. The review concluded that AKG had alleviative effect on oxidative stress by two pathways, which were antioxidative enzymes activities and nonenzymatic oxidative decarboxylation in hydrogen peroxide decomposition, respectively. In fact, AKG mainly acted as a source of energy and an antioxidant in the process of antioxidation. Additionally, AKG developed its antioxidative functions on various clinical diseases in animals and humans, such as burns, trauma, postoperative recovery, and aged diseases. Furthermore, it also raised that whether AKG could directly activate Nrf2/ARE signaling pathway to alleviate oxidative stress or not. The review well summarized the antioxidative function of AKG and provided a good reference for AKG in practical applications in animals and humans.

L. Yang et al. described the fact that proanthocyanidins (PCs) could be used against oxidative stress (OS); PCs have been found to prevent OS damage by downregulating the reactive oxygen species (ROS) and OS-induced DNA damage and promoted DNA repair by different pathways: (i) scavenging oxidative species (e.g., ROS and RNS) and free radicals, thereby disturbing direct OS damage and redox chain reactions, (ii) enhancing the functions of DNA repair enzymes, (ii) dose-dependent inhibiting cyclobutane pyridine dimers (CPD) formation, (iv) rapidly repairing CPDs through the induction of IL-12, (v) promoting the nucleotide excision repair mechanism, and (vi) inhibiting DNA hypomethylation; they reduced lipid peroxidation and modulated different signaling pathways (e.g., MAPK, NF-кB, Nrf2, and PI3k/Akt) involved in OS. Finally, PCs could be readily affordable without side effects compared with other synthetic compounds against OS.

F. He et al. reviewed the functions and signaling mechanisms of amino acids in intestinal inflammation. Amino acids, including essential amino acids (EAAs), conditionally essential amino acids (CEAAs), and nonessential amino acids (NEAAs), improve the functions of intestinal barrier, increase expressions of anti-inflammatory cytokines, and tight junction proteins but decrease oxidative stress and the apoptosis of enterocytes as well as the expressions of proinflammatory cytokines in the intestinal inflammation. The functions of amino acids in intestinal inflammation are associated with various signaling pathways, including mechanistic target of rapamycin (mTOR), inducible nitric oxide synthase (iNOS), calcium-sensing receptor (CaSR), nuclear factor-kappa-B (NF-*κ*B), mitogen-activated protein kinase (MAPK), nuclear erythroid-related factor 2 (Nrf2), general controlled nonrepressed kinase 2 (GCN2), and angiotensin-converting enzyme 2 (ACE2).

P. Huang et al. described a significant decrease in the severity of hepatic inflammation and ameliorated hepatic fibrosis in nonalcoholic steatohepatitis (NASH) rats, via the pathological examination and real-time quantitative PCR detecting system (QPCR), after the treatment of a novel liver-specific liver X receptor (LXR) inverse agonist, SR9243. SR9243 treatment significantly decreased liver fibrosis induced by bile-duct ligation (BDL) and carbon tetrachloride (CCL4) in NASH rats and exerted a certain inhibitory effect on the level of insulin, total cholesterol, increased liver enzymes, and the level of CD68, TNF-*α*, IL-1*β*, and IL-6 which can increase hepatic inflammatory injury. The result of this article shows a significant therapeutic potential in treating NASH with LXR inverse agonist SR9243.

D. Zhu et al. described melatonin (MEL) impact on the antioxidant capacity and intestinal bacteria community in 5% DSS-induced mouse model. MEL treatment improved significantly the antioxidant capability in relative to the DSS group. In addition,* Bacteroidetes* were the most abundant phylum in the DSS group (58.93%), followed by* Firmicutes* with 31.46% and* Proteobacteria* with 7.97%. In contrast,* Firmicutes* were the most abundant in the MEL group (49.48%), followed by* Bacteroidetes* with 41.63% and* Proteobacteria* with 7.50%. But there no differences between two groups in diversity index, bacterial culture abundance, and coverage. This study indicated that melatonin could improve internal health by enhancing oxidative stress resistance and ameliorating intestinal microbial flora.

W. Song et al. described the fact that the aim of this study was to determine the effects of ethanol extract of* Ulva prolifera* (EUP) on insulin tolerance, inflammatory cytokines secretion, and oxidative status in high-fat-diet- (HFD-) treated mice; EUP supplementation protected mice from HFD-induced body weight gain and improved glucose sensitivity and insulin resistance. Additionally, EUP supplementation lowered reactive oxygen species concentration, while enhancing glutathione level and glutathione peroxidase activity in HFD-treated mice; this study suggested that EUP might have the potential effects on the prevention of metabolic disease.

P. Bin et al. described that the oxidation resistance of methionine and cysteine, two of the most representative sulfur amino acids. Methionine exerted its antioxidant capacity by methionine residues and SAM. Methionine residues are prone to be oxidized by diversified forms of ROS, and SAM increases the activity of enzyme increasing the GSH level. Similar to the methionine, cysteine exerted its antioxidant capacity by cysteine residues and its metabolites (GSH, H_2_S, and taurine), cysteine residues are easily reacted with H_2_O_2_, and the products of cysteine are reported to alleviate oxidant stress induced by various oxidants and protect the tissue from the damage.

In conclusion, we expect that our special issue updates scientific reports in current research and presents useful thoughts for the readers.

